# Tetra­kis(μ-2-phen­oxy­propionato)-κ^3^ *O*,*O*′:*O*′;κ^3^ *O*:*O*,*O*′,κ^4^ *O*:*O*′-bis­[(1,10-phenanthroline-κ^2^ *N*,*N*′)(2-phen­oxy­propionato-κ^2^ *O*,*O*′)dysprosium(III)]

**DOI:** 10.1107/S1600536811034696

**Published:** 2011-08-31

**Authors:** Jin-Bei Shen, Jia-Lu Liu, Guo-Liang Zhao

**Affiliations:** a College of Chemistry and Life Sciences, Zhejiang Normal University, Jinhua 321004, Zhejiang, People’s Republic of China; b Zhejiang Normal University Xingzhi College, Jinhua, Zhejiang 321004, People’s Republic of China

## Abstract

In the centrosymmetric binuclear title complex, [Dy_2_(C_9_H_9_O_3_)_6_(C_12_H_8_N_2_)_2_], the two Dy^III^ ions are linked by four 2-phen­oxy­propionate (*L*) groups through their bi- and tridentate bridging modes. Each Dy^III^ ion is nine-coordinated by one 1,10-phenanthroline mol­ecule, one bidentate carboxyl­ate group and four bridging carboxyl­ate groups in a distorted DyN_2_O_7_ monocapped square-anti­prismatic geometry. The title compound is isotypic with its terbium-containing analogue.

## Related literature

For the terbium analogue of the title compound, see: Shen *et al.* (2011 ▶). For a related structure, see: Liu *et al.* (2010 ▶).
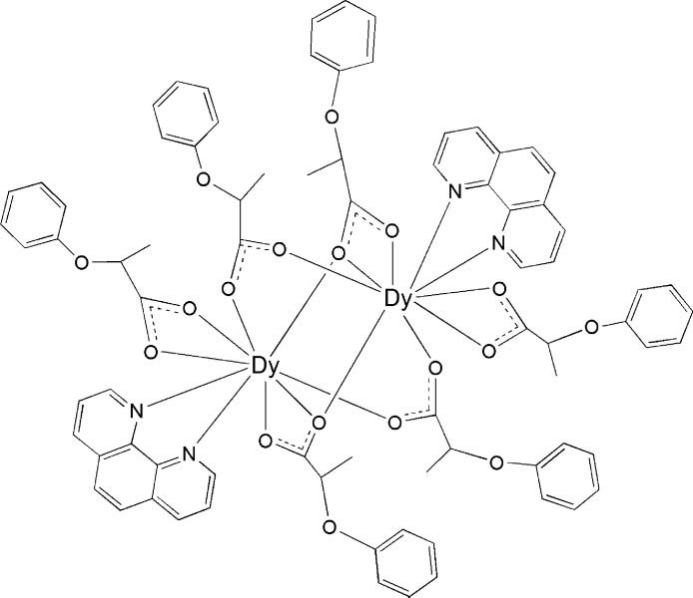


## Experimental

### 

#### Crystal data

[Dy_2_(C_9_H_9_O_3_)_6_(C_12_H_8_N_2_)_2_]
*M* *_r_* = 1676.38
Monoclinic, 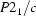

*a* = 11.4738 (1) Å
*b* = 25.8057 (3) Å
*c* = 13.8525 (2) Å
β = 120.657 (1)°
*V* = 3528.32 (7) Å^3^
*Z* = 2
Mo *K*α radiation
μ = 2.18 mm^−1^
*T* = 296 K
0.32 × 0.14 × 0.08 mm

#### Data collection

Bruker APEXII CCD diffractometer
Absorption correction: multi-scan (*SADABS*; Sheldrick, 1996 ▶) *T*_min_ = 0.710, *T*_max_ = 0.840
47810 measured reflections
6224 independent reflections
5057 reflections with *I* > 2σ(*I*)
*R*_int_ = 0.043

#### Refinement

*R*[*F* ^2^ > 2σ(*F* ^2^)] = 0.025
*wR*(*F* ^2^) = 0.053
*S* = 1.05
6224 reflections
463 parameters
H-atom parameters constrained
Δρ_max_ = 0.76 e Å^−3^
Δρ_min_ = −0.49 e Å^−3^

### 

Data collection: *APEX2* (Bruker, 2006 ▶); cell refinement: *SAINT* (Bruker, 2006 ▶); data reduction: *SAINT*; program(s) used to solve structure: *SHELXS97* (Sheldrick, 2008 ▶); program(s) used to refine structure: *SHELXL97* (Sheldrick, 2008 ▶); molecular graphics: *SHELXTL* (Sheldrick, 2008 ▶); software used to prepare material for publication: *SHELXL97*.

## Supplementary Material

Crystal structure: contains datablock(s) I, global. DOI: 10.1107/S1600536811034696/hb6365sup1.cif

Structure factors: contains datablock(s) I. DOI: 10.1107/S1600536811034696/hb6365Isup2.hkl

Additional supplementary materials:  crystallographic information; 3D view; checkCIF report

## Figures and Tables

**Table 1:** Selected bond lengths (Å)

Dy1—O8^i^	2.3252 (18)
Dy1—O4	2.3328 (18)
Dy1—O5^i^	2.3731 (19)
Dy1—O1	2.412 (2)
Dy1—O7	2.4421 (19)
Dy1—O2	2.462 (2)
Dy1—N2	2.518 (2)
Dy1—N1	2.594 (2)
Dy1—O8	2.622 (2)

Symmetry code: (i) 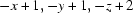.

**Fig. 1.**
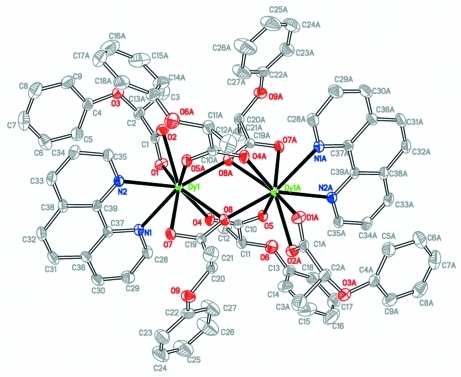
The molecular structure of (I) with displacement ellipsoids drawn at the 30% probability level.

**Fig. 2.**
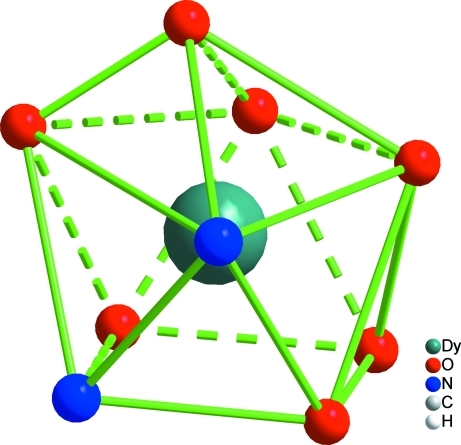
The coordination environment of the Dy(III) ion.

## supplementary crystallographic information

### Comment

As part of our ongoing studies of 2-phenoxypropionic acid complexes (Shen *et al.*, 2011) we describe a new Dy^III^ complex.

The structure of the title compound (1) is a dinuclear dysprosium complex with Dy—Dy separation of 3.9857 (3) Å. The structure of the complex (Fig. 1) reveals that the molecule contains six *L*, two phen molecules and two Dy^III^ ions. Each Dy(III) ion is coordinated to nine atoms, of which five oxygen atoms are from the bridging carboxylates, two oxygen atoms from the bidentate chelating carboxylate group, and two nitrogen atoms from a 1,10- phenanthroline molecule. The *L* ligands are coordinated to the Dy^III^ ions in three different modes: chelating,bridging and bridging tridentate.The analysis of structural features indicates that the central Dy(III) ion adopts a distorted monocapped square antiprism geometry(Fig. 2).The Dy—O distances are all within the range 2.3252 (18)–2.622 (2) Å, and the Dy—N distances rang from 2.518 (2)–2.594 (2) Å, all of which are within the range of those of other nine-coordinated Dy^III^ complexes with carboxylic donor ligands and 1,10- phenanthroline (Liu *et al.*, 2010). The selected bond lengths and angles for complex 1 are listed in Table 1. In addition, there are no classical hydrogen bonds in the crystal structure, because good hydrogen bond donors are absent. The most significant intermolecular interactions are C—H···O hydrogen bonds (Table 2) and weak π···π aromatic interactions from phen molecules and aromatic rings of the *L* ligands.

### Experimental

Reagents and solvents used were of commercially available quality and without purified before using. 2-phenoxypropionic acid (1.5 mmol), Dy(NO_3_)_3_.5H_2_O (0.5 mmol) and 1,10-phenanthroline (0.5 mmol) were dissolved in 20 ml e nthanol, then 10 ml water was added to the above solution. The mixed solution was stirred for 12 h at room temperature. At last, deposit was filtered out and the colourless solution was kept in the open air. Colourless blocks of (I) were obtained after several days.

### Refinement

The structure was solved by direct methods and successive Fourier difference synthesis. The H atoms bonded to C and N atoms were positioned geometrically and refined using a riding model [aliphatic C—H =0.96 Å (*U*_iso_(H) = 1.5*U*_eq_(C)), aromatic C—H = 0.93 Å (*U*_iso_(H) = 1.2*U*_eq_(C))].

### Crystal data

**Table:**

[Dy_2_(C_9_H_9_O_3_)_6_(C_12_H_8_N_2_)_2_]	*F*(000) = 1684
*M**_r_* = 1676.38	*D*_x_ = 1.578 Mg m^−^^3^
Monoclinic, *P*2_1_/*c*	Mo *K*α radiation, λ = 0.71073 Å
Hall symbol: -P 2ybc	Cell parameters from 9890 reflections
*a* = 11.4738 (1) Å	θ = 1.6–25.0°
*b* = 25.8057 (3) Å	µ = 2.18 mm^−^^1^
*c* = 13.8525 (2) Å	*T* = 296 K
β = 120.657 (1)°	Block, colourless
*V* = 3528.32 (7) Å^3^	0.32 × 0.14 × 0.08 mm
*Z* = 2	

### Data collection

**Table:**

Bruker APEXII CCD diffractometer	6224 independent reflections
Radiation source: fine-focus sealed tube	5057 reflections with *I* > 2σ(*I*)
graphite	*R*_int_ = 0.043
φ and ω scans	θ_max_ = 25.0°, θ_min_ = 1.6°
Absorption correction: multi-scan (*SADABS*; Sheldrick, 1996)	*h* = −13→13
*T*_min_ = 0.710, *T*_max_ = 0.840	*k* = −30→30
47810 measured reflections	*l* = −16→16

### Refinement

**Table:**

Refinement on *F*^2^	Primary atom site location: structure-invariant direct methods
Least-squares matrix: full	Secondary atom site location: difference Fourier map
*R*[*F*^2^ > 2σ(*F*^2^)] = 0.025	Hydrogen site location: inferred from neighbouring sites
*wR*(*F*^2^) = 0.053	H-atom parameters constrained
*S* = 1.05	*w* = 1/[σ^2^(*F*_o_^2^) + (0.0204*P*)^2^ + 2.0937*P*] where *P* = (*F*_o_^2^ + 2*F*_c_^2^)/3
6224 reflections	(Δ/σ)_max_ = 0.002
463 parameters	Δρ_max_ = 0.76 e Å^−^^3^
0 restraints	Δρ_min_ = −0.49 e Å^−^^3^

### Special details

**Table:**

Geometry. All e.s.d.'s (except the e.s.d. in the dihedral angle between two l.s. planes) are estimated using the full covariance matrix. The cell e.s.d.'s are taken into account individually in the estimation of e.s.d.'s in distances, angles and torsion angles; correlations between e.s.d.'s in cell parameters are only used when they are defined by crystal symmetry. An approximate (isotropic) treatment of cell e.s.d.'s is used for estimating e.s.d.'s involving l.s. planes.
Refinement. Refinement of *F*^2^ against ALL reflections. The weighted *R*-factor *wR* and goodness of fit *S* are based on *F*^2^, conventional *R*-factors *R* are based on *F*, with *F* set to zero for negative *F*^2^. The threshold expression of *F*^2^ > σ(*F*^2^) is used only for calculating *R*-factors(gt) *etc*. and is not relevant to the choice of reflections for refinement. *R*-factors based on *F*^2^ are statistically about twice as large as those based on *F*, and *R*- factors based on ALL data will be even larger.

### Fractional atomic coordinates and isotropic or equivalent isotropic displacement parameters (Å^2^)

**Table:**

	*x*	*y*	*z*	*U*_iso_*/*U*_eq_	
Dy1	0.453434 (12)	0.502684 (5)	0.840438 (10)	0.02670 (5)	
O1	0.6186 (2)	0.51253 (8)	0.78321 (18)	0.0434 (5)	
O2	0.5317 (2)	0.58487 (8)	0.80244 (17)	0.0396 (5)	
O3	0.5945 (2)	0.63358 (8)	0.65590 (18)	0.0525 (6)	
O4	0.5818 (2)	0.43014 (7)	0.93621 (16)	0.0378 (5)	
O6	0.8286 (2)	0.34859 (9)	1.15437 (19)	0.0545 (6)	
O7	0.24603 (19)	0.45817 (7)	0.80147 (15)	0.0351 (5)	
O8	0.38341 (19)	0.46859 (7)	0.98205 (15)	0.0347 (5)	
O9	0.0919 (2)	0.38935 (8)	0.84166 (18)	0.0428 (5)	
N1	0.3810 (2)	0.43228 (9)	0.68656 (19)	0.0324 (6)	
N2	0.2821 (2)	0.53041 (9)	0.64369 (19)	0.0327 (6)	
C1	0.6015 (3)	0.56069 (13)	0.7717 (2)	0.0366 (7)	
C2	0.6729 (3)	0.59088 (13)	0.7208 (3)	0.0461 (8)	
H2A	0.6912	0.5677	0.6741	0.055*	
C3	0.8052 (4)	0.61361 (16)	0.8136 (3)	0.0701 (12)	
H3A	0.8499	0.6317	0.7810	0.105*	
H3B	0.8626	0.5862	0.8607	0.105*	
H3C	0.7867	0.6373	0.8576	0.105*	
C4	0.4858 (3)	0.62449 (13)	0.5497 (3)	0.0445 (8)	
C5	0.4495 (4)	0.57701 (14)	0.4988 (3)	0.0523 (9)	
H5A	0.4959	0.5473	0.5373	0.063*	
C6	0.3424 (4)	0.57372 (17)	0.3886 (3)	0.0648 (11)	
H6A	0.3185	0.5417	0.3527	0.078*	
C7	0.2717 (4)	0.6172 (2)	0.3327 (3)	0.0696 (12)	
H7A	0.1996	0.6148	0.2593	0.084*	
C8	0.3082 (4)	0.66421 (18)	0.3855 (4)	0.0695 (12)	
H8A	0.2607	0.6938	0.3474	0.083*	
C9	0.4138 (4)	0.66828 (14)	0.4940 (3)	0.0570 (10)	
H9A	0.4369	0.7003	0.5297	0.068*	
C10	0.6565 (3)	0.41422 (10)	1.0358 (2)	0.0310 (7)	
C11	0.7397 (3)	0.36640 (12)	1.0443 (3)	0.0409 (8)	
H11A	0.6765	0.3383	1.0024	0.049*	
C12	0.8235 (4)	0.37670 (15)	0.9910 (3)	0.0615 (10)	
H12A	0.8721	0.3458	0.9941	0.092*	
H12B	0.8868	0.4041	1.0309	0.092*	
H12C	0.7653	0.3867	0.9142	0.092*	
C13	0.7780 (4)	0.31777 (12)	1.2062 (3)	0.0483 (9)	
C14	0.6469 (4)	0.31529 (13)	1.1806 (3)	0.0580 (10)	
H14A	0.5802	0.3334	1.1192	0.070*	
C15	0.6133 (5)	0.28599 (16)	1.2454 (5)	0.0848 (14)	
H15A	0.5238	0.2845	1.2282	0.102*	
C16	0.7106 (8)	0.25915 (19)	1.3345 (5)	0.1028 (19)	
H16A	0.6872	0.2396	1.3785	0.123*	
C17	0.8423 (7)	0.26066 (17)	1.3603 (4)	0.0971 (18)	
H17A	0.9079	0.2415	1.4204	0.117*	
C18	0.8778 (4)	0.29032 (14)	1.2974 (3)	0.0677 (12)	
H18A	0.9677	0.2921	1.3155	0.081*	
C19	0.2736 (3)	0.45226 (10)	0.9000 (2)	0.0284 (6)	
C20	0.1737 (3)	0.42670 (11)	0.9256 (2)	0.0351 (7)	
H20A	0.2227	0.4098	0.9992	0.042*	
C21	0.0777 (3)	0.46674 (14)	0.9259 (3)	0.0513 (9)	
H21A	0.0140	0.4502	0.9412	0.077*	
H21B	0.0300	0.4833	0.8538	0.077*	
H21C	0.1283	0.4922	0.9827	0.077*	
C22	0.1558 (3)	0.34625 (12)	0.8335 (3)	0.0464 (9)	
C23	0.0787 (4)	0.31532 (14)	0.7417 (3)	0.0624 (11)	
H23A	−0.0101	0.3246	0.6897	0.075*	
C24	0.1336 (5)	0.27023 (16)	0.7269 (4)	0.0831 (14)	
H24A	0.0814	0.2494	0.6645	0.100*	
C25	0.2633 (5)	0.25611 (16)	0.8029 (4)	0.0919 (16)	
H25A	0.3003	0.2260	0.7925	0.110*	
C26	0.3375 (5)	0.28667 (17)	0.8938 (5)	0.1056 (19)	
H26A	0.4250	0.2766	0.9472	0.127*	
C27	0.2861 (4)	0.33248 (15)	0.9089 (4)	0.0812 (15)	
H27A	0.3400	0.3537	0.9700	0.097*	
C28	0.4289 (3)	0.38448 (12)	0.7054 (3)	0.0426 (8)	
H28A	0.5034	0.3769	0.7756	0.051*	
C29	0.3745 (4)	0.34462 (13)	0.6264 (3)	0.0553 (10)	
H29A	0.4127	0.3117	0.6436	0.066*	
C30	0.2646 (3)	0.35470 (13)	0.5238 (3)	0.0503 (9)	
H30A	0.2263	0.3285	0.4704	0.060*	
C31	0.0942 (3)	0.41847 (14)	0.3935 (3)	0.0485 (9)	
H31A	0.0510	0.3931	0.3388	0.058*	
C32	0.0467 (3)	0.46716 (14)	0.3716 (3)	0.0454 (8)	
H32A	−0.0284	0.4749	0.3020	0.055*	
C33	0.0656 (3)	0.55867 (13)	0.4337 (3)	0.0433 (8)	
H33A	−0.0074	0.5684	0.3645	0.052*	
C34	0.1306 (3)	0.59444 (13)	0.5165 (3)	0.0447 (8)	
H34A	0.1040	0.6290	0.5040	0.054*	
C35	0.2374 (3)	0.57857 (12)	0.6203 (2)	0.0383 (7)	
H35A	0.2798	0.6034	0.6765	0.046*	
C36	0.2097 (3)	0.40457 (12)	0.4990 (3)	0.0398 (8)	
C37	0.2721 (3)	0.44260 (11)	0.5833 (2)	0.0313 (7)	
C38	0.1095 (3)	0.50737 (12)	0.4535 (2)	0.0368 (7)	
C39	0.2203 (3)	0.49463 (11)	0.5605 (2)	0.0316 (7)	
O5	0.66605 (19)	0.43284 (7)	1.12245 (15)	0.0339 (5)	

### Atomic displacement parameters (Å^2^)

**Table:**

	*U*^11^	*U*^22^	*U*^33^	*U*^12^	*U*^13^	*U*^23^
Dy1	0.02533 (8)	0.02954 (8)	0.02117 (7)	0.00035 (6)	0.00892 (6)	0.00075 (6)
O1	0.0494 (14)	0.0361 (13)	0.0527 (14)	0.0023 (10)	0.0320 (12)	0.0056 (10)
O2	0.0366 (13)	0.0382 (12)	0.0437 (13)	−0.0001 (10)	0.0202 (11)	0.0038 (10)
O3	0.0620 (16)	0.0452 (14)	0.0407 (13)	−0.0113 (11)	0.0193 (12)	0.0073 (10)
O4	0.0455 (13)	0.0359 (11)	0.0262 (11)	0.0119 (10)	0.0140 (10)	0.0033 (9)
O6	0.0462 (15)	0.0519 (14)	0.0520 (15)	0.0093 (11)	0.0153 (12)	0.0090 (11)
O7	0.0322 (12)	0.0440 (12)	0.0247 (11)	−0.0062 (9)	0.0114 (9)	−0.0027 (9)
O8	0.0288 (11)	0.0389 (12)	0.0263 (11)	−0.0038 (9)	0.0067 (9)	−0.0005 (9)
O9	0.0275 (12)	0.0396 (13)	0.0488 (13)	−0.0041 (10)	0.0105 (10)	0.0008 (10)
N1	0.0328 (14)	0.0361 (14)	0.0268 (13)	0.0017 (11)	0.0140 (11)	0.0000 (10)
N2	0.0316 (14)	0.0359 (14)	0.0276 (13)	−0.0011 (11)	0.0128 (11)	0.0004 (11)
C1	0.0288 (17)	0.049 (2)	0.0237 (16)	−0.0053 (15)	0.0078 (14)	0.0047 (14)
C2	0.046 (2)	0.051 (2)	0.0406 (19)	−0.0046 (16)	0.0222 (17)	0.0091 (16)
C3	0.046 (2)	0.101 (3)	0.052 (2)	−0.023 (2)	0.0169 (19)	0.016 (2)
C4	0.048 (2)	0.052 (2)	0.0347 (18)	−0.0060 (16)	0.0219 (17)	0.0071 (15)
C5	0.054 (2)	0.055 (2)	0.045 (2)	0.0009 (18)	0.0238 (18)	0.0013 (17)
C6	0.060 (3)	0.083 (3)	0.050 (2)	−0.010 (2)	0.027 (2)	−0.019 (2)
C7	0.049 (2)	0.117 (4)	0.039 (2)	−0.002 (3)	0.0195 (19)	0.009 (2)
C8	0.056 (3)	0.081 (3)	0.066 (3)	0.008 (2)	0.027 (2)	0.030 (2)
C9	0.061 (3)	0.050 (2)	0.061 (2)	−0.0015 (18)	0.031 (2)	0.0117 (18)
C10	0.0313 (17)	0.0276 (15)	0.0307 (17)	−0.0014 (12)	0.0133 (14)	0.0010 (12)
C11	0.0383 (19)	0.0416 (18)	0.0350 (17)	0.0110 (14)	0.0130 (15)	0.0021 (14)
C12	0.050 (2)	0.071 (3)	0.069 (3)	0.0174 (19)	0.035 (2)	0.007 (2)
C13	0.064 (3)	0.0311 (18)	0.051 (2)	0.0070 (17)	0.031 (2)	0.0046 (15)
C14	0.065 (3)	0.038 (2)	0.069 (3)	0.0000 (18)	0.033 (2)	0.0031 (18)
C15	0.108 (4)	0.052 (3)	0.124 (4)	−0.005 (3)	0.081 (4)	−0.003 (3)
C16	0.183 (7)	0.060 (3)	0.101 (4)	0.001 (4)	0.098 (5)	0.013 (3)
C17	0.144 (5)	0.059 (3)	0.053 (3)	0.004 (3)	0.025 (3)	0.018 (2)
C18	0.073 (3)	0.041 (2)	0.064 (3)	0.0025 (19)	0.016 (2)	0.0033 (19)
C19	0.0257 (16)	0.0265 (15)	0.0291 (16)	0.0027 (12)	0.0113 (14)	−0.0003 (12)
C20	0.0312 (17)	0.0406 (17)	0.0306 (16)	−0.0025 (14)	0.0137 (14)	0.0023 (13)
C21	0.047 (2)	0.063 (2)	0.058 (2)	0.0026 (18)	0.0369 (19)	0.0013 (18)
C22	0.038 (2)	0.0361 (18)	0.054 (2)	−0.0052 (15)	0.0153 (17)	0.0058 (15)
C23	0.057 (2)	0.055 (2)	0.058 (2)	−0.0035 (19)	0.017 (2)	−0.0062 (19)
C24	0.094 (4)	0.054 (3)	0.075 (3)	−0.009 (2)	0.024 (3)	−0.019 (2)
C25	0.092 (4)	0.049 (3)	0.111 (4)	0.009 (2)	0.034 (3)	−0.019 (3)
C26	0.075 (3)	0.058 (3)	0.130 (4)	0.022 (2)	0.013 (3)	−0.020 (3)
C27	0.055 (3)	0.048 (2)	0.091 (3)	0.0095 (19)	0.001 (2)	−0.016 (2)
C28	0.044 (2)	0.0434 (19)	0.0331 (17)	0.0058 (15)	0.0142 (15)	−0.0015 (14)
C29	0.067 (3)	0.0393 (19)	0.056 (2)	0.0042 (17)	0.029 (2)	−0.0084 (16)
C30	0.055 (2)	0.048 (2)	0.043 (2)	−0.0107 (17)	0.0217 (18)	−0.0172 (16)
C31	0.047 (2)	0.063 (2)	0.0309 (18)	−0.0149 (18)	0.0162 (16)	−0.0160 (16)
C32	0.039 (2)	0.067 (2)	0.0246 (17)	−0.0079 (17)	0.0119 (15)	−0.0001 (16)
C33	0.0367 (19)	0.057 (2)	0.0293 (17)	0.0050 (16)	0.0117 (15)	0.0118 (15)
C34	0.045 (2)	0.047 (2)	0.0380 (18)	0.0113 (16)	0.0185 (16)	0.0110 (15)
C35	0.0413 (19)	0.0390 (18)	0.0293 (16)	0.0033 (14)	0.0142 (15)	0.0022 (13)
C36	0.0402 (19)	0.0454 (19)	0.0343 (17)	−0.0115 (15)	0.0194 (15)	−0.0095 (14)
C37	0.0316 (17)	0.0387 (17)	0.0282 (16)	−0.0058 (13)	0.0185 (14)	−0.0044 (13)
C38	0.0293 (16)	0.055 (2)	0.0247 (15)	−0.0046 (14)	0.0127 (13)	0.0020 (14)
C39	0.0297 (16)	0.0407 (17)	0.0253 (14)	−0.0014 (13)	0.0148 (13)	0.0027 (13)
O5	0.0376 (12)	0.0351 (11)	0.0260 (11)	0.0050 (9)	0.0141 (9)	0.0009 (9)

### Geometric parameters (Å, °)

**Table:**

Dy1—O8^i^	2.3252 (18)	C13—C14	1.359 (5)
Dy1—O4	2.3328 (18)	C13—C18	1.391 (5)
Dy1—O5^i^	2.3731 (19)	C14—C15	1.370 (5)
Dy1—O1	2.412 (2)	C14—H14A	0.9300
Dy1—O7	2.4421 (19)	C15—C16	1.358 (7)
Dy1—O2	2.462 (2)	C15—H15A	0.9300
Dy1—N2	2.518 (2)	C16—C17	1.365 (7)
Dy1—N1	2.594 (2)	C16—H16A	0.9300
Dy1—O8	2.622 (2)	C17—C18	1.368 (6)
Dy1—Dy1^i^	3.9857 (3)	C17—H17A	0.9300
O1—C1	1.256 (4)	C18—H18A	0.9300
O2—C1	1.249 (4)	C19—C20	1.514 (4)
O3—C4	1.379 (4)	C20—C21	1.511 (4)
O3—C2	1.417 (4)	C20—H20A	0.9800
O4—C10	1.265 (3)	C21—H21A	0.9600
O6—C13	1.382 (4)	C21—H21B	0.9600
O6—C11	1.411 (4)	C21—H21C	0.9600
O7—C19	1.243 (3)	C22—C27	1.364 (5)
O8—C19	1.263 (3)	C22—C23	1.375 (5)
O8—Dy1^i^	2.3252 (18)	C23—C24	1.388 (5)
O9—C22	1.368 (4)	C23—H23A	0.9300
O9—C20	1.431 (3)	C24—C25	1.364 (6)
N1—C28	1.321 (4)	C24—H24A	0.9300
N1—C37	1.362 (3)	C25—C26	1.356 (6)
N2—C35	1.320 (4)	C25—H25A	0.9300
N2—C39	1.360 (4)	C26—C27	1.384 (5)
C1—C2	1.537 (4)	C26—H26A	0.9300
C2—C3	1.520 (5)	C27—H27A	0.9300
C2—H2A	0.9800	C28—C29	1.396 (4)
C3—H3A	0.9600	C28—H28A	0.9300
C3—H3B	0.9600	C29—C30	1.360 (5)
C3—H3C	0.9600	C29—H29A	0.9300
C4—C5	1.369 (5)	C30—C36	1.397 (4)
C4—C9	1.380 (5)	C30—H30A	0.9300
C5—C6	1.391 (5)	C31—C32	1.341 (5)
C5—H5A	0.9300	C31—C36	1.430 (4)
C6—C7	1.370 (5)	C31—H31A	0.9300
C6—H6A	0.9300	C32—C38	1.432 (4)
C7—C8	1.368 (6)	C32—H32A	0.9300
C7—H7A	0.9300	C33—C34	1.361 (4)
C8—C9	1.371 (5)	C33—C38	1.393 (4)
C8—H8A	0.9300	C33—H33A	0.9300
C9—H9A	0.9300	C34—C35	1.393 (4)
C10—O5	1.245 (3)	C34—H34A	0.9300
C10—C11	1.528 (4)	C35—H35A	0.9300
C11—C12	1.504 (5)	C36—C37	1.410 (4)
C11—H11A	0.9800	C37—C39	1.437 (4)
C12—H12A	0.9600	C38—C39	1.414 (4)
C12—H12B	0.9600	O5—Dy1^i^	2.3731 (19)
C12—H12C	0.9600		
			
O8^i^—Dy1—O4	73.74 (7)	C7—C8—C9	120.9 (4)
O8^i^—Dy1—O5^i^	77.87 (7)	C7—C8—H8A	119.6
O4—Dy1—O5^i^	134.95 (7)	C9—C8—H8A	119.6
O8^i^—Dy1—O1	88.29 (7)	C8—C9—C4	119.5 (4)
O4—Dy1—O1	84.10 (7)	C8—C9—H9A	120.2
O5^i^—Dy1—O1	129.42 (7)	C4—C9—H9A	120.2
O8^i^—Dy1—O7	123.51 (7)	O5—C10—O4	127.1 (3)
O4—Dy1—O7	90.56 (7)	O5—C10—C11	119.5 (2)
O5^i^—Dy1—O7	76.83 (7)	O4—C10—C11	113.3 (3)
O1—Dy1—O7	144.82 (7)	O6—C11—C12	107.3 (3)
O8^i^—Dy1—O2	76.51 (7)	O6—C11—C10	115.2 (3)
O4—Dy1—O2	128.28 (7)	C12—C11—C10	110.5 (3)
O5^i^—Dy1—O2	75.89 (7)	O6—C11—H11A	107.9
O1—Dy1—O2	53.54 (7)	C12—C11—H11A	107.9
O7—Dy1—O2	141.10 (7)	C10—C11—H11A	107.9
O8^i^—Dy1—N2	144.82 (7)	C11—C12—H12A	109.5
O4—Dy1—N2	139.62 (7)	C11—C12—H12B	109.5
O5^i^—Dy1—N2	79.44 (7)	H12A—C12—H12B	109.5
O1—Dy1—N2	85.71 (8)	C11—C12—H12C	109.5
O7—Dy1—N2	76.07 (7)	H12A—C12—H12C	109.5
O2—Dy1—N2	72.09 (7)	H12B—C12—H12C	109.5
O8^i^—Dy1—N1	147.16 (7)	C14—C13—O6	126.4 (3)
O4—Dy1—N1	75.53 (7)	C14—C13—C18	120.1 (4)
O5^i^—Dy1—N1	133.69 (7)	O6—C13—C18	113.3 (4)
O1—Dy1—N1	77.28 (7)	C13—C14—C15	119.9 (4)
O7—Dy1—N1	67.74 (7)	C13—C14—H14A	120.1
O2—Dy1—N1	115.25 (7)	C15—C14—H14A	120.1
N2—Dy1—N1	64.11 (7)	C16—C15—C14	120.1 (5)
O8^i^—Dy1—O8	72.80 (7)	C16—C15—H15A	119.9
O4—Dy1—O8	69.56 (7)	C14—C15—H15A	119.9
O5^i^—Dy1—O8	69.02 (6)	C15—C16—C17	120.6 (5)
O1—Dy1—O8	150.83 (7)	C15—C16—H16A	119.7
O7—Dy1—O8	51.08 (6)	C17—C16—H16A	119.7
O2—Dy1—O8	137.15 (7)	C16—C17—C18	119.9 (5)
N2—Dy1—O8	122.48 (7)	C16—C17—H17A	120.0
N1—Dy1—O8	106.73 (7)	C18—C17—H17A	120.0
O8^i^—Dy1—C1	83.52 (8)	C17—C18—C13	119.3 (5)
O4—Dy1—C1	107.99 (9)	C17—C18—H18A	120.4
O5^i^—Dy1—C1	102.64 (9)	C13—C18—H18A	120.4
O1—Dy1—C1	26.90 (8)	O7—C19—O8	121.7 (3)
O7—Dy1—C1	151.28 (7)	O7—C19—C20	120.8 (2)
O2—Dy1—C1	26.79 (8)	O8—C19—C20	117.5 (2)
N2—Dy1—C1	75.64 (8)	O7—C19—Dy1	56.71 (14)
N1—Dy1—C1	95.25 (9)	O8—C19—Dy1	65.02 (15)
O8—Dy1—C1	155.99 (7)	C20—C19—Dy1	177.16 (19)
O8^i^—Dy1—C19	98.53 (7)	O9—C20—C21	106.6 (2)
O4—Dy1—C19	79.17 (7)	O9—C20—C19	111.3 (2)
O5^i^—Dy1—C19	71.26 (7)	C21—C20—C19	110.0 (2)
O1—Dy1—C19	159.31 (7)	O9—C20—H20A	109.6
O7—Dy1—C19	25.19 (7)	C21—C20—H20A	109.6
O2—Dy1—C19	147.06 (7)	C19—C20—H20A	109.6
N2—Dy1—C19	99.19 (8)	C20—C21—H21A	109.5
N1—Dy1—C19	86.74 (8)	C20—C21—H21B	109.5
O8—Dy1—C19	25.89 (6)	H21A—C21—H21B	109.5
C1—Dy1—C19	172.83 (9)	C20—C21—H21C	109.5
O8^i^—Dy1—Dy1^i^	38.94 (5)	H21A—C21—H21C	109.5
O4—Dy1—Dy1^i^	66.85 (5)	H21B—C21—H21C	109.5
O5^i^—Dy1—Dy1^i^	69.01 (4)	C27—C22—O9	125.0 (3)
O1—Dy1—Dy1^i^	123.85 (5)	C27—C22—C23	119.6 (3)
O7—Dy1—Dy1^i^	84.77 (4)	O9—C22—C23	115.4 (3)
O2—Dy1—Dy1^i^	110.26 (5)	C22—C23—C24	119.8 (4)
N2—Dy1—Dy1^i^	146.11 (6)	C22—C23—H23A	120.1
N1—Dy1—Dy1^i^	132.80 (5)	C24—C23—H23A	120.1
O8—Dy1—Dy1^i^	33.87 (4)	C25—C24—C23	120.6 (4)
C1—Dy1—Dy1^i^	122.35 (6)	C25—C24—H24A	119.7
C19—Dy1—Dy1^i^	59.65 (5)	C23—C24—H24A	119.7
C1—O1—Dy1	92.75 (19)	C26—C25—C24	118.9 (4)
C1—O2—Dy1	90.56 (18)	C26—C25—H25A	120.6
C4—O3—C2	118.7 (3)	C24—C25—H25A	120.6
C10—O4—Dy1	139.55 (18)	C25—C26—C27	121.5 (4)
C13—O6—C11	119.1 (3)	C25—C26—H26A	119.2
C19—O7—Dy1	98.10 (16)	C27—C26—H26A	119.2
C19—O8—Dy1^i^	162.47 (19)	C22—C27—C26	119.5 (4)
C19—O8—Dy1	89.09 (16)	C22—C27—H27A	120.3
Dy1^i^—O8—Dy1	107.20 (7)	C26—C27—H27A	120.3
C22—O9—C20	117.3 (2)	N1—C28—C29	123.9 (3)
C28—N1—C37	117.2 (3)	N1—C28—H28A	118.0
C28—N1—Dy1	124.38 (19)	C29—C28—H28A	118.0
C37—N1—Dy1	117.64 (18)	C30—C29—C28	119.0 (3)
C35—N2—C39	117.5 (2)	C30—C29—H29A	120.5
C35—N2—Dy1	121.60 (19)	C28—C29—H29A	120.5
C39—N2—Dy1	120.41 (18)	C29—C30—C36	119.7 (3)
O2—C1—O1	122.5 (3)	C29—C30—H30A	120.2
O2—C1—C2	119.2 (3)	C36—C30—H30A	120.2
O1—C1—C2	118.3 (3)	C32—C31—C36	121.6 (3)
O2—C1—Dy1	62.65 (16)	C32—C31—H31A	119.2
O1—C1—Dy1	60.35 (16)	C36—C31—H31A	119.2
C2—C1—Dy1	173.9 (2)	C31—C32—C38	121.1 (3)
O3—C2—C3	105.9 (3)	C31—C32—H32A	119.4
O3—C2—C1	111.8 (3)	C38—C32—H32A	119.4
C3—C2—C1	110.1 (3)	C34—C33—C38	119.4 (3)
O3—C2—H2A	109.7	C34—C33—H33A	120.3
C3—C2—H2A	109.7	C38—C33—H33A	120.3
C1—C2—H2A	109.7	C33—C34—C35	119.1 (3)
C2—C3—H3A	109.5	C33—C34—H34A	120.5
C2—C3—H3B	109.5	C35—C34—H34A	120.5
H3A—C3—H3B	109.5	N2—C35—C34	123.9 (3)
C2—C3—H3C	109.5	N2—C35—H35A	118.1
H3A—C3—H3C	109.5	C34—C35—H35A	118.1
H3B—C3—H3C	109.5	C30—C36—C37	117.6 (3)
C5—C4—O3	125.0 (3)	C30—C36—C31	123.3 (3)
C5—C4—C9	120.4 (3)	C37—C36—C31	119.1 (3)
O3—C4—C9	114.5 (3)	N1—C37—C36	122.7 (3)
C4—C5—C6	119.1 (3)	N1—C37—C39	117.9 (2)
C4—C5—H5A	120.4	C36—C37—C39	119.4 (3)
C6—C5—H5A	120.4	C33—C38—C39	118.0 (3)
C7—C6—C5	120.5 (4)	C33—C38—C32	123.2 (3)
C7—C6—H6A	119.7	C39—C38—C32	118.9 (3)
C5—C6—H6A	119.7	N2—C39—C38	122.1 (3)
C8—C7—C6	119.5 (4)	N2—C39—C37	118.0 (2)
C8—C7—H7A	120.3	C38—C39—C37	119.8 (3)
C6—C7—H7A	120.3	C10—O5—Dy1^i^	134.66 (18)

Symmetry codes: (i) −*x*+1, −*y*+1, −*z*+2.
